# Silk Bioconjugates: From Chemistry and Concept to Application

**DOI:** 10.1021/acsbiomaterials.2c01116

**Published:** 2023-01-27

**Authors:** Saphia A. L. Matthew, F. Philipp Seib

**Affiliations:** † Strathclyde Institute of Pharmacy and Biomedical Sciences, University of Strathclyde, 161 Cathedral Street, Glasgow G4 0RE, U.K.; ‡ Branch Bioresources, Fraunhofer Institute for Molecular Biology and Applied Ecology, Ohlebergsweg 12, 35392 Giessen, Germany

**Keywords:** silk fibroin, spidroin, chemistry, polymer therapeutics, bioconjugation

## Abstract

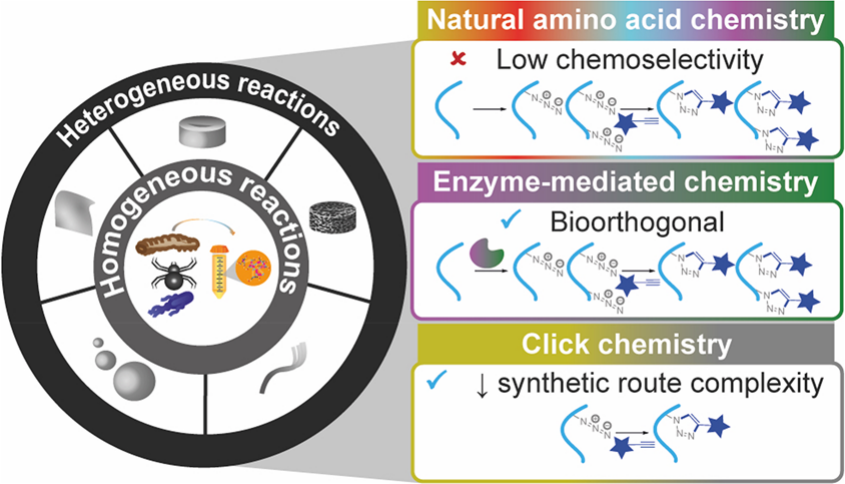

Medical silks have captured global interest. While silk sutures have a long track record in humans, silk bioconjugates are still in preclinical development. This perspective examines key advances in silk bioconjugation, including the fabrication of silk–protein conjugates, bioconjugated silk particles, and bioconjugated substrates to enhance cell–material interactions in two and three dimensions. Many of these systems rely on chemical modification of the silk biopolymer, often using carbodiimide and reactive ester chemistries. However, recent progress in enzyme-mediated and click chemistries has expanded the molecular toolbox to enable biorthogonal, site-specific conjugation in a single step when combined with recombinant silk fibroin tagged with noncanonical amino acids. This perspective outlines key strategies available for chemical modification, compares the resulting silk conjugates to clinical benchmarks, and outlines open questions and areas that require more work. Overall, this assessment highlights a domain of new sunrise capabilities and development opportunities for silk bioconjugates that may ultimately offer new ways of delivering improved healthcare.

## 1 Introduction

We are familiar with mulberry silk for its use in clothing and perhaps with the use of silk as a traditional suture material.^1^ However, silk has far wider potential.^2^ We and others typically use the term silk to refer to protein-based fiber-forming materials spun by living organisms. In this terminology, we also include silk-inspired proteins produced by recombinant approaches.^3,4^ However, the domesticated silkworm *Bombyx mori* is the most common everyday silk source, and all clinically approved silk products are based on this silk.^2,5^ The use of *B. mori* silk for biomedical applications is motivated by key silk hallmarks, including biocompatibility, biodegradability, mechanical properties, and the ability to “unspin” the silk fiber to yield an all-aqueous regenerated silk fibroin stock (detailed below).^2,5,6^ This regenerated silk can be processed under mild conditions into new material formats (e.g., films, scaffolds, hydrogels, particles, etc.).^7^

Several silk products are licensed for medical use in humans and have navigated the global regulatory landscape (e.g., Regulation (EU) 2017/745 to obtain CE marking analogous to the Class III Premarket Approval/510(k) in the USA).^2,5^ For example, in the early 2000s, AlPreTec Srl (San Donà di Piave, Italy) launched the first commercial silk medical device covalently functionalized with 3-trimethylsilylpropyl-dimethyloctadecyl ammonium chloride (i.e., DermaSilk); this modified silk was recently reformulated as a powder, Epifibroin 0039, for topical wound care and dermatological conditions. Epifibroin 0039 received Class IIb CE marking in 2019 and is out licensed to MediSilk Spa (San Donà di Piave, Italy).^8^ However, most publicly available reports on the biocompatibility of silk in humans come from silk sutures (reviewed in ref (1)) and SERI Surgical Scaffold. SERI Surgical Scaffold obtained 510(k) clearance by the FDA in 2008, was launched on the market in 2013 (reviewed in ref (9)), and was discontinued in December 2021 because of off-label use and associated side effects in some individuals.^5^ The regulatory path for native silk and silk bioconjugate products has recently been reviewed.^5^

For the purpose of this perspective, “bioconjugates” are broadly defined to include polymer–biologics or scaffold–biologics conjugates and genetically engineered biologics. A broad range of polymers is used to create this large family of “bioconjugates.” Within this family, the synthetic polymer poly(ethylene glycol) (PEG) is the most successful and has been a critical enabler of the translation of an ever-increasing product portfolio into routine clinical use, now dating back more than 30 years.^10^ Since the first clinical PEGylated product, PEG-adenosine deaminase (Adagen, Enzo), was marketed in 1990, PEGylation (detailed below) technologies have grown this product family to 20 marketed PEGylated proteins plus one biosimilar product, pegfilgrastim (Fulphila).^10,11^ Clearly, PEGylation and the molecular tool box available for protein expression have been keys to clinical success. This foundation not only informs the future generation of polymer protein conjugates but also delivers impact further afield (detailed below).

We will use this state-of-the-art as the backdrop when examining progress made with silk-based bioconjugates. This perspective will specifically focus on the biopolymer silk, which has many favorable properties (detailed below). By including examples using mulberry and non-mulberry silks, spidroins, and recombinant silks as feedstocks, we will provide selected examples of polymer– and scaffold-–iologics conjugates, the chemical modification and genetic engineering tools of silks to create these conjugates, the associated challenges, and emerging trends. We provide an overview, and a critical analysis of selected key studies that have created silk bioconjugates. When possible, we point to comprehensive reviews to complement this perspective, which provides examples and is by no means a complete review of the literature. We will conclude with a future perspective and highlight some of the key questions that the silk community faces when developing silk bioconjugates.

## 2 Medical Silk: Fundamentals

In contrast to native spun silk products (i.e., silk sutures, SERI Surgical Scaffold, and silk garments), many of the emerging biomedical applications are based on reconstituted silk and recombinant spider-like silks that open up many opportunities but also bring their own unique sets of challenges (for timely reviews, see refs (3, 4, and 12)). The potential for clinical translation is now evidenced by the approval of the first reconstituted *B. mori* silk fibroin injectable (Silk Voice, Sofregen Inc., Medford, MA, USA) for vocal fold augmentation in 2019. Here, regenerated silk fibroin, i.e., liquid silk, was processed into “particles” with a microsized porous scaffold structure. These particles were embedded within a hyaluronic acid hydrogel and filled into a patented applicator for injection.^13^ The Silk Voice product has demonstrated that reconstituted silk fibroin can be acceptable for registration in medical regulatory frameworks. This is important, and more products are likely to emerge. For example, an ongoing clinical trial (NCT04085822) sponsored by Silk Medical Aesthetics Inc. (Medford, MA, USA) is examining the use of this technology to improve aesthetic appearance in humans, while small-scale clinical trials using fibroin formulated as silk films^14^ and sponges^15^ have also shown favorable outcomes for both wound repair and aesthetics. Silk Biomaterials Srl (Lomazzo, Italy) is developing a more complex hybrid all-silk medical device called SilkBridge to serve as a nerve conduit. SilkBridge is a three-layered tubular scaffold; the core consists of a silk fibroin braided yarn textile and is coated with electrospun silk both inside and outside.^16^ Overall, the clinically approved products, the emerging pipeline, and a clinical workforce familiar with silk provide the necessary foundation for next-generation silk products.^2,5^

The silk protein degrades *in vitro* and *in vivo*.^17^ However, degradation depends on a multitude of factors, such as the silk amount, secondary structure, material format, and implantation site.^17^ Predicting silk degradation is challenging because simple sequence alignment to proteolytic enzymes does not match lab measurements, indicating the importance of factors beyond the primary sequence, such as protein folding and thus enzyme accessibility to the cleavage site.^18^ Improved biodegradation tools are emerging and should help bridge this gap.^19^ Silk degradation has been reviewed in detail elsewhere,^20^ and pure silk fibroin is typically regarded as nonimmunogenic.^1,2^ However, the impact of silk, and its biodegradation products, on the immune system requires more work, especially for reconstituted silk formats. First studies using single cell RNA sequencing approaches now provide the necessary resolution to uncover complex cell–silk dynamics,^21,22^ while noninvasive bioluminescence *in vivo* studies,^23^ human blood compatibility,^24−26^ corona formation,^27^ and metabolic studies^28,29^ expand our understanding of the immune response toward silk and novel silk formats. These studies typically use *B. mori* silk, but seminal work by Hanna Dams-Kozlowska and co-workers examined the *in vivo* immune response of recombinantly expressed spider silk-like particles.^30^ These types of studies are important to de-risk new materials and formats.

## 3 Silk Structure, Source, and Processing

**Figure 1:**
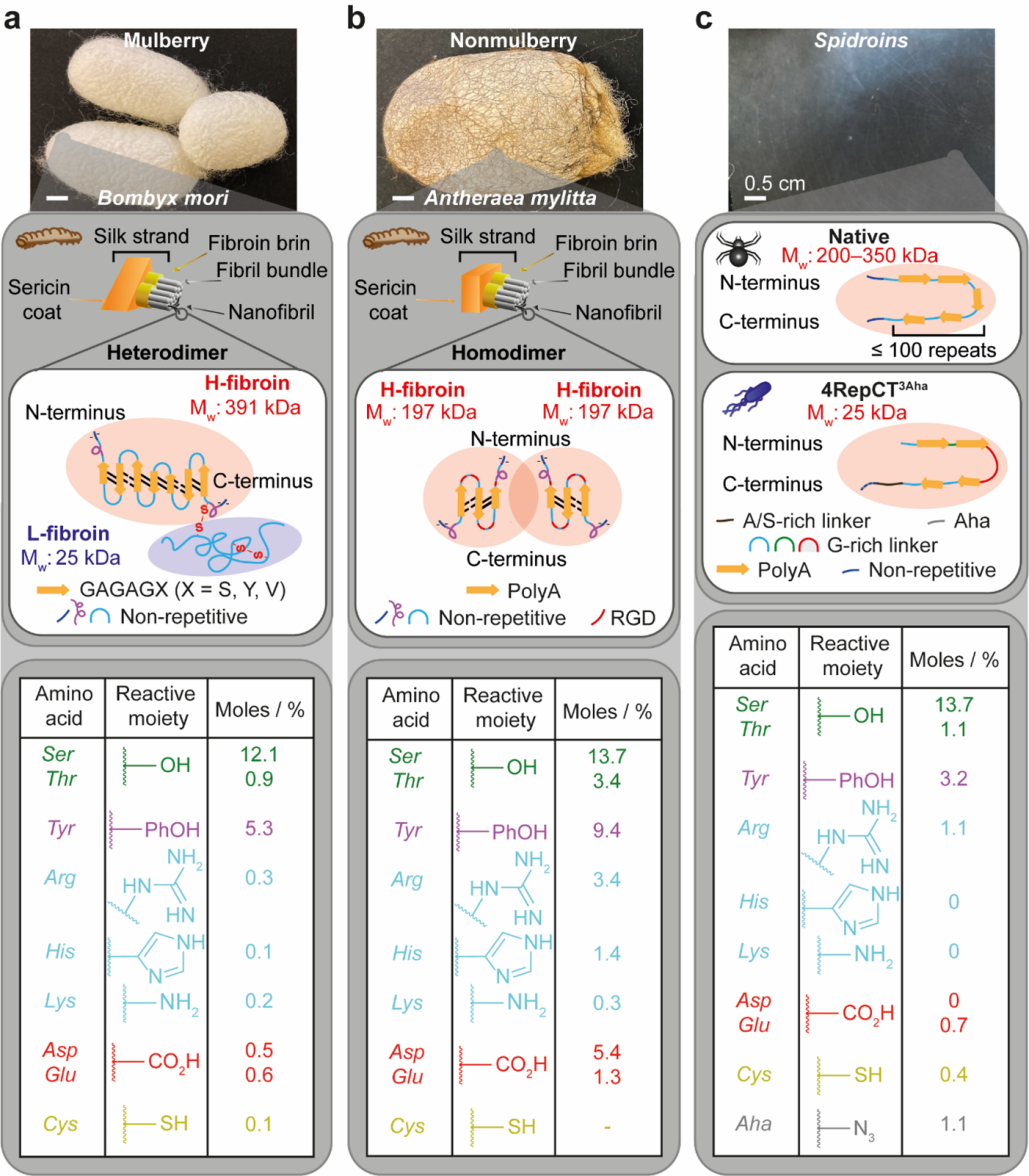
Schematic representations and the reactive amino acid composition of (a) *Bombyx mori* silk fibroin, (b) *Antheraea mylitta* silk fibroin, and (c) native and recombinant spidroins. The scale bars are 0.5 cm in length. The structural composition and reactive amino acid content of silk fibroin have been assembled from refs (36, 39−42).

The biopolymer silk is made of amino acids, but the sequence and overall structure are origin dependent.^31^ Silk fibroin proteins across different insect species are characterized by a heavy chain and share many common features^32,33^ (Figure 1). Mulberry silk from *B. mori* is widely used to create bioconjugates and has thus been examined in greater detail. The *B. mori* silk protein is modular and consists of light (≈26 kDa) and heavy (≈391 kDa) chains that are linked by a single disulfide bond at the C-terminus^34^ (Figure 1a). The light chain has a nonrepeating amino acid sequence, whereas the heavy chain features C-terminal and N-terminal capping sequences as the only completely nonrepeating amino acid residues. However, advances in genome sequencing and population-scale functional genomics studies open up new insights into genomic variants, core genes, and nonredundant structure variations introduced during sericulture selection, ultimately impacting the silk sequence and quality.^35^

Many mulberry and non-mulberry silk fibroin properties arise due to the block copolymer-like arrangement of the silk heavy chain. For example, the *B. mori* heavy chain contains 11 short hydrophilic regions and 12 hydrophobic blocks that account for 94% of the silk heavy chain. These hydrophobic blocks contain predominately glycine-X (GX) repeats, where X is alanine (A) (65%), serine (S) (23%), or tyrosine (Y) (9%).^2,34^ In contrast, the repeating sequences of the non-mulberry silk (e.g., *Antheraea mylitta*) heavy chain alternate between hydrophilic serine-aspartic acid-serine and hydrophobic polyalanine repeats (Figure 1b). Non-mulberry silk also contains the integrin-binding arginylglycylaspartic acid (RGD) sequence, which provides a potential advantage over *B. mori* silk in biomedical applications where enhanced cell adhesion is desired without the need for additional silk modifications^36^ (Figure 1b).

Spider silks, known as “spidroins”, also have a block copolymer composition but lack the heavy and light chain configuration typically found in insect silks (Figure 1c). Across the spider kingdom, variations in the spidroin sequence enable spiders to thrive in diverse habitats.^33^ Unlike *B. mori*, spiders produce several different silk types via dedicated silk glands.^4^ Advances in transcriptome assembly and new open-source spider silk data sets^37^ change our ability to mine and identify new silk-inspired materials. Multiplexing these data rich resources (e.g., refs (35, 37, 38)) using machine learning tools is expected to create novel silks. Traditionally, the drag line silks from the gold orb-web spider (*Nephila clavipes*) and the European garden spider (*Araneus diadematus*) are the most commonly studied (reviewed in refs (3 and 4)). Unlike *B. mori* sericulture, spiders cannot be farmed because of their territorial and cannibalistic behavior. Therefore, recombinant expression systems are used to produce “spider silk-inspired” proteins (often denoted as “mini-spidroins”^4^).

Mini-spidroins are isolated using standard molecular tools to yield a pristine protein.^4^ Pristine *B. mori* and *A. mylitta* silk fibroin can also be obtained when isolated directly from the silk gland (e.g., refs (43 and 44)). Indeed, most studies extract *A. mylitta* silk directly as the highly stable polyalanine β-sheets formed after spinning impart a low solubility in most solvents.^36^ However, for *B. mori* silk fibroin, isolation of the protein from the spun cocoon has become a standard technique due to the greater ease of extraction compared to silk gland dissection; see ref (7) for a detailed description of common protocols.

During cocoon spinning, the silk thread is encased in sericin as it emerges from the spinneret of the silkworm. This sericin coat acts as a glue during cocoon construction. Sericin is currently being explored for biomedical applications with promising results.^45^ However, silk with sericin is attributed to induce an inflammatory response, and sericin is therefore removed by a process known as “degumming”.^2^ A broad spectrum of degumming techniques has been developed using chemical, enzymatic, and physical techniques which result in varying degrees of chain scission of the fibroin backbone. The most popular process boils the chopped silk cocoon in a weakly alkaline solution.^7^ The degummed silk fibers are then rinsed with water and dried to yield a polydisperse mixture of silk fibroin polypeptides.

Many studies also “unspin” these fibers with the use of either the organic solvent hexafluoroisopropanol or aqueous chaotropic agents at high concentrations and elevated temperatures. These treatments dissolve higher-order structures by disrupting hydrogen bonding and van der Waals interactions of the β sheet assemblies to turn solid silk II into a liquid silk I state but can also reduce the average molecular weight.^46−48^ Next, the aqueous liquid silk is dialyzed against water to remove the chaotropic salt. The resulting aqueous silk fibroin solution, also termed regenerated silk, is used for downstream processing (e.g., chemical modification).

## 4 Bioconjugation through Natural Amino Acid Chemistry

**Figure 2:**
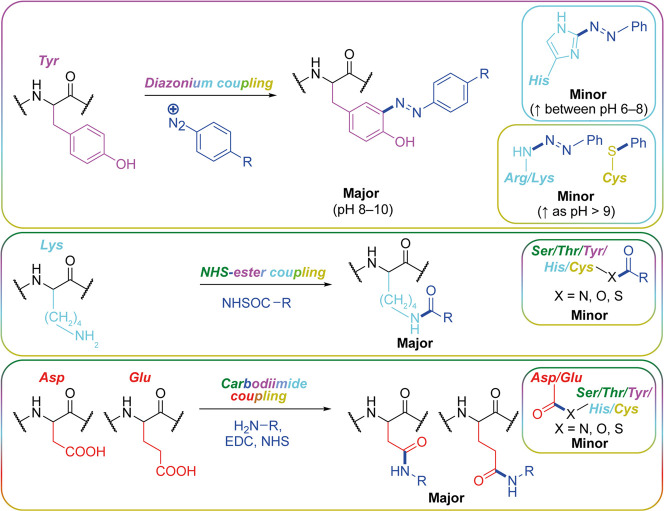
Popular bioconjugation techniques which display a range of chemoselectivities and utilize the reactive silk fibroin natural amino acid chemistry. The reaction schemes have been adapted from refs (49 and 51−53).

Chemical modification of silk fibroin exploits the natural amino acids bearing side groups with electrophilic and nucleophilic moieties (Figure 2). These chemical modifications have been expertly reviewed elsewhere^41,49^ and are also applicable for other protein molecules.^50^

### 4.1 Homogeneous Reactions

Homogeneous reactions for chemical modification of liquid silk substrates provide a valuable synthetic route to a range of material formats and morphologies. However, due to the noncovalent interactions and protein folding which protects reactive amino acids from incoming reactants in aqueous solvents, solution-phase reactions can suffer from limitations including complicated purifications from unreacted substrate and low reaction yields.

**Figure 3:**
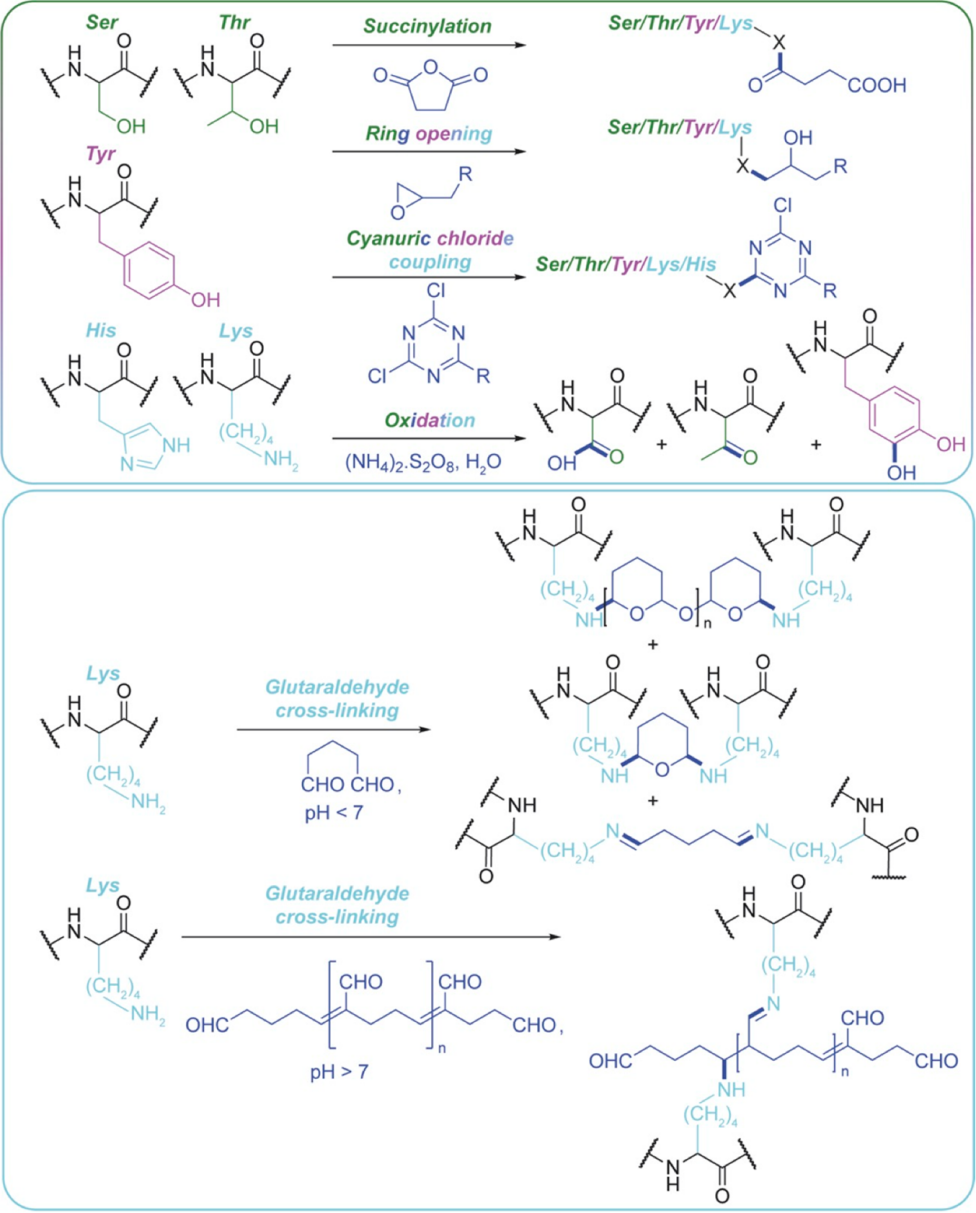
Bioconjugation techniques which display low chemoselectivity and utilize the reactive silk fibroin natural amino acid chemistry. The reaction schemes have been adapted from refs (41, 49, 53, and 71−74).

To increase the conjugation efficiency of carboxyl groups in solution phase, the Burke lab reported an anhydrous succinylation strategy.^54^ Degummed silk was dissolved under anhydrous conditions in ionic liquid, followed by dimethylformamide treatment to reduce the solution viscosity.^54^ While surfactants helped to open up silk hydrophobic regions, the use of 8 M urea worked best by fully disrupting hydrogen bonding. Succinic anhydride was used to modify the hydroxyl groups of the serine and threonine residues and the amine groups of the lysine, arginine, and histidine residues (Figure 3). The use of urea doubled the substitution degree of carboxylic acid functional groups to 90% overall. Despite these chemical modifications and a lower overall β-sheet content, β-sheet formation was still possible and solution-stable silk films and bulk materials were generated. However, chemical modification reduced the zeta potential, which had a direct impact on adsorptive drug loading by increasing electrostatic drug loading and greater cumulative drug release. Carboxylic acid-functionalized liquid silk samples were also used for carbodiimide conjugation using dopamine as a model compound. The inclusion of a surfactant in the reaction conditions doubled the reaction yields to a 65% degree of substitution.^54^ The degrees of carboxyl substitution were characterized using ^1^H NMR and conductometric titrations while dopamine functionalization was quantified by ^1^H NMR. The low abundance of histidine (5), lysine (12), and arginine (14) residues makes it difficult to verify their successful modification and stoichiometry due to the masking effects of substantially higher numbers of threonine (47) and serine residues (635). However, this study demonstrated that opening up the solution state of silk provides access to typically shielded locations of the silk protein to maximize the efficiency of carboxylation reactions. These reactive handles can then be exploited for bioconjugation.

An alternative approach to increase silk carboxyl content exploiting the tyrosine functionality only was piloted by Serban and colleagues.^55^ Liquid silk fibroin was chemically modified to generate silk-based ionomers carrying either polylysine or polyglutamic acid charges. The conjugation used carbodiimide-mediated coupling; however, to maximize the grafting densities, the carboxylic acid content was chemically increased by reacting the tyrosine residues with diazonium salt and chloroacetic acid to hydroxylate the serine residues. This starting silk stock solution was then used for polylysine or polyglutamic acid conjugation. Colloidal composites could be generated by mixing the ionomeric pair at high concentrations (i.e., 25% w/v), while combining them at lower concentrations (i.e., 5% w/v). This self-assembly was driven by electrostatic interactions and was pH dependent and reversible. Experimental measurements suggested that these network assemblies appeared to be polarized, with the interacting poly(amino acid) chains clustered in the core of the particles and the silk backbone oriented outward. These ionomers supported cell growth and could be loaded with drugs, opening up their potential biomedical use.^55^ This chemistry was subsequently used to synthesize a library of functionalized silk polyelectrolytes that also included a double-brush design. The silk conjugates contained poly(l-lysine), poly(l-glutamic acid), and PEG side chains with different grafting architectures. Fine tuning of the poly(amino acid) lengths and of the molecular weight and degree of PEG grafting enabled the successful layer-by-layer encapsulation of Gram-negative and Gram-positive bacteria engineered to act as biosensors. The high PEG grafting and the double-brush design were particularly effective in enabling the formation of hydrogen bonding shells that were cytocompatible and protected the encapsulated cells for up to 4 months under aqueous ambient conditions.^56^ However, the overall reaction yields or conjugation efficiencies remain unknown. In follow-up work, poly(l-lysine) silk, poly(l-glutamic acid)-crafted silks, and poly(l-lysine)-*block*-PEG silk were used to form pH-responsive microcapsules.^57^ Several other studies beyond the scope of the present bioconjugate perspective have also described the carboxylation of silk for use in wet adhesion using dual silk networks^58^ and the tuning hydrogen bonding capacity for improved tuning of silk adhesion.^59^

Diazonium coupling for the modification of tyrosine residues (and to a lesser extent histidine, as it is only present at 0.18 mol %) has also been used to introduce azide functional groups into liquid silk.^60^ The diazonium reaction was monitored using absorbance spectroscopy, and the yield was approximately 50%. The azide silk was then subjected to biorthogonal Cu(I)-catalyzed cycloaddition to covalently attach alkyne-functionalized PEG at 88% click chemistry efficiency. The silk was then allowed to dry into films that were methanol-treated to induce beta sheets. The aqueous solution-stable films functionalized with PEG showed great hydrophilicity.^60^ Although click chemistry was first described more than a century ago,^61^ this chemistry has only come to the fore over the past decade. Exemplary work by the Murphy lab has now established a reaction sequence to control and monitor tyrosine modification.^62^ Here, the native tyrosine was converted into azobenzene, followed by reduction of the azo bond to yield an amino-tyrosine group that can be acetylated under mild conditions with carboxylic acid, *N*-hydroxysuccinimide-ester derivatives, or alkyne and azide derivatives suitable for click chemistry. The advantages of this amino-tyrosine group synthesis are the introduction of a nucleophilic amine, the absence of the highly colored azo group that interferes with analytical methods, and the excellent control provided for monitoring the reaction yield using 2D NMR in combination with stable isotope labeling.^62^ The use of naturally reactive amino acids and their derivatives to introduce click handles can open up multistep synthetic routes due to the highly specific and efficient nature of biorthogonal click chemistry.^50^

Motta and colleagues used click chemistry to synthesize dual-functional silk to create basement mimetics with both laminin IKVAV peptide and collagen functionality.^63^ First, silk fibroin was functionalized with azide groups using diazonium coupling. This azide-modified silk was then used to conduct 1-ethyl-3-(3-(dimethylamino)propyl)carbodiimide (EDC) *N*-hydroxysuccinimide (NHS) mediated conjugation of the IKVAV peptide. Next, the IKVAV-functionalized azide silk was subjected to an azide–alkyne cycloaddition reaction to covalently attach collagen IV to the silk. Prior to this click chemistry, in separate sets of reactions, the collagen was modified with alkyne functionality. Overall, this silk conjugation strategy produced dual-functional silk that was cast into films or self-assembled into physically cross-linked hydrogels. Cell adhesion studies showed improved cell proliferation for these basement membrane-inspired silk mimetics.^63^ These dual-function substrates were inspired by previous work by the same group.^64^

Here, the Motta lab used self-assembled *B. mori* silk hydrogels that were bioconjugated with IKVAV to improve human neural stem cell cultures.^64^ The IKVAV pentapeptide derived from laminin was covalently attached to silk using standard carbodiimide chemistry. Sonication energy was used to drive a 1% w/w silk solution to the gel transition. These self-assembled silk hydrogels had a stiffness of 258 Pa, closely mimicking brain mechanics. Primarily cell-based assays were used to prove the successful conjugation. The 3D encapsulation of cells within these IKVAV-functionalized hydrogels significantly improved cell proliferation and enhanced neuronal differentiation markers.^64^

The creation of silk films with covalently conjugated biologically active macromolecules has also been reported. For example, silk films with interferon gamma and interleukin 4 were used to modulate macrophage responses.^65^ Macrophage polarization into putative inflammatory (M1) and anti-inflammatory (M2) phenotypes is important for pathogen defense and wound repair, respectively. However, a chronic switch from the resting to an activated phenotype is associated with disease.^66^ Therefore, controlling macrophage responses locally is appealing to improve tissue engineering outcomes. Reeves and colleagues tuned interferon gamma and interleukin 4 release from silk films to direct macrophages toward an M1- and M2-like phenotype. Short-term biomacromolecule release was tuned using silk crystallinity via bulk material dissolution, whereas the 10 day long-term release used covalently immobilized interferon with a sacrificial disulfide bond. First, 4-(2-aminoethyl)-aniline was conjugated to tyrosines using liquid silk to increase the number of primary amines. These primary amine groups were then reacted with sulfosuccinimidyl 6-[30-(2-pyridyldithio)propionamido]hexanoate) to create an activated silk fibroin. Interferon does not contain cysteine and therefore has no endogenous free sulfhydryl groups that can be used to form a disulfide bond. Therefore, interferon was reacted with Traut’s reagent to create a sulfhydryl group that was then reacted with the activated silk fibroin film to form a silk disulfide–interterferon conjugate. Exposure of the conjugate to phosphate buffered saline triggered a biphasic interferon release with a burst release (60%) of payload during the first 24 h, followed by a slow release in the remaining 9 days. The biological activity of the released interferon was assessed by the CCR7n and CD206 gene expression.^65^ One of the key challenges when conjugating these biologically active proteins is to ensure that the protein retains its native configuration and that the released protein is devoid of any residual linkers; this information was absent.

Another example of a biologically active silk bioconjugate is silk immobilized heparin. Heparin-functionalized silk films were used to bind and release vascular endothelial growth factor, as well as to modify the hemocompatibility of the film.^25^ For some samples, heparin was covalently linked to soluble silk using standard EDC/NHS carbodiimide chemistry. Here, a 28-fold molar excess of carboxylic acid functional groups on heparin relative to the primary amine groups of silk resulted in functionalization of 59% of the available reactive sites and produced a conjugate that was 2.1% heparin by weight (reaction yield 85%). Films were generated by solution casting, and beta sheets were induced to make them solution stable. *In vitro* human blood compatibility studies showed an initial inflammatory response, irrespective of the heparin presentation mode. However, covalently conjugated heparin showed the lowest blood coagulation response and outperformed the reference material, polytetrafluoroethylene, that is used clinically.^25^

#### 4.1.1 Polymer–Protein Conjugates

Polymer–protein conjugates are one of the most successful nanomedicines, with clinical approval dating back more than 30 years and 20 products marketed currently.^10,11^ The motivation for conjugating polymers to proteins is to improve their pharmacokinetics by increasing residence time in blood (or tissue), thereby reducing dosing frequency, improving solubility and physical and chemical stability, and reducing protein aggregation, proteolytic degradation, and immune activation. The gold standard is PEG, with both linear and branched configurations.^10^ Protein polymers are being explored as PEG alternatives; the most notable of these is poly(glutamic acid), which has reached Phase III human clinical trials.^67^ Silk-protein conjugates have also been reported in preclinical studies exploring both solid and liquid silks.

A liquid silk fibroin–l-asparaginase conjugate was created using glutaraldehyde-mediated conjugation.^68^ Reaction conditions were screened, including cross-linker concentration, molar ratio, pH, reaction time, and temperature, to yield silk–l-asparaginase conjugates. The product was isolated using anion exchange (Q Sepharose FF) and gel filtration (Sephacryl S-300 HR) chromatography. Under optimized conditions, the ε-amino group modification was 58%, while the modified l-asparaginase retained 67% of its original enzymatic activity. The silk fibroin-modified asparaginase had improved thermal stability, especially at high temperatures (60–80 °C) and substantially improved resistance to proteolytic degradation (up to 25%).^68^

Work by Kenji Kiguchi and co-workers^69^ synthesized superior silk fibroin-modified asparaginase conjugates. Silk fibroin was solution conjugated to asparaginase using glutaraldehyde chemistry under optimized reaction conditions at a 5 mL scale.^69^ Here, 0.05 M phosphate buffer pH 7.4 was used containing 50 mg of liquid silk fibroin, 5 mg of asparaginase, and 0.5% glutaraldehyde in the presence of 2.5 mg of l-asparagine, which served as an active-center protector for the enzyme. The reaction was kept at 4 °C and allowed to react for 10 h. The resulting lead formulation of silk fibroin and asparaginase (10:1) retained 80% of its enzymatic activity and significantly improved storage stability, with 80% activity remaining after 30 days, compared to 20% for the unmodified enzyme. Temperature stressing at 60 °C doubled the enzymatic activity, which remained at >80%, although at 70 °C, it dropped to 20% like the unmodified enzyme. The silk fibroin conjugate showed great protease stability compared to unmodified asparaginase (63 and 33 h half-life, respectively). Protease stability could be further improved by increasing the cross-linking time, but this came at the cost of asparaginase activity; the optimized balance was found to be 10 h of cross-linking. Unlike any other studies, this work also generated Lineweaver–Burk plots and determined the Michaelis constant as approximately 6 times lower than the unmodified asparaginase kinetics, indicating that the apparent substrate affinity was increased for the silk fibroin conjugate.^69^ This is remarkable and has since been reported with enzymes physically embedded in silk solution and solid silk samples.^70^ The current working hypothesis is that the silk nanocrystalline regions have four key stabilizing effects: a buffering action; a tailoring of water content at the nanoscale; provision of physical protection; and reduction of payload mobility.

The work by Kenji Kiguchi and co-workers also determined immunological response *in vivo* by counter immunoelectrophoresis to detect antigen and antibody complexes directed toward asparaginase immunity.^69^ The immune priming study used New Zealand White rabbits divided into three groups receiving silk fibroin (20 mg), asparaginase–silk conjugate, or asparaginase alone (control; 2 mg of enzyme equivalents) in complete Freud’s adjuvant and injected repeatedly over several weeks using intraperitoneal, subcutaneous, and intravenous administration routes. The conjugated silk fibroin significantly reduced the immunological response, as also verified by immune precipitation over a wide concentration range, because the silk fibroin–asparaginase conjugate showed very low levels of asparaginase-directed antibody reactivity compared to the unmodified asparaginase enzyme.^69^ This low immunological response is very encouraging and has implications beyond the present study. However, several key challenges remain (detailed below); one is the low specificity of glutaraldehyde chemistry (Figure 3) (plus concerns about the potential toxicity of residual cross-linkers).

Decorating silk fibroin with biomacromolecules using glutaraldehyde or EDC/NHS chemistries lacks specificity, resulting in the potential cross-linking of the biomacromolecule. Seminal work by Lorenz Meinel and colleagues compared EDC/NHS with Cu(I)-catalyzed azide–alkyne cycloaddition (CuAAC; click chemistry) to conjugate Fibroblast Growth Factor 2 to silk.^75^ Comparison of the product characteristics of the bioconjugates revealed that both conjugation strategies converted endogenous tyrosine residues to azido or carboxylated functional groups using diazonium coupling chemistry. A broad spectrum of molar equivalents of diazonium salts to tyrosine residues was explored, ranging from 1 to 100%. The carboxylated aqueous silks were stable for >60 days at 4 °C, whereas azido silks up to 5 mol % and 100% were typically stable for a few days but only for minutes at intermediate mole percentages. Perhaps the high degree of modification impaired β sheet assembly due to structural changes in self-assembly, while at a low degree of modification, the solution-stable silk conformation was retained. Secondary structure analysis showed that β sheet formation was responsible for silk fibroin solution–gel transition. Reactive dyes were used to verify the successful conversion of the azido or carboxylated functional groups in solution. These dyes worked well at low molar ratios, but the 50 and 100 mol % carboxylated silks precipitated from solution. By contrast, click chemistry samples worked across the entire substitution spectrum. The suitability of the conjugation chemistry was demonstrated using Fibroblast Growth Factor 2 conjugation. To improve receptor engagement, a 3 kg/mol PEG spacer was introduced between the silk fibroin and Fibroblast Growth Factor 2. Both click chemistry and EDC conjugation were successful in creating PEGylated silk fibroin–Fibroblast Growth Factor 2 conjugates. The key advantage of this click chemistry strategy is its fidelity, which ensures that only the growth factor is tethered in the absence of growth factor cross-linking, resulting in covalent conjugates. Eliminating Fibroblast Growth Factor 2 cross-linking is important because it can evoke an immune response that could cross react to impact endogenously produced Fibroblast Growth Factor 2 and trigger unintended pharmacological responses in the host. The biological response toward silk fibroin/Fibroblast Growth Factor conjugates remains to be determined.^75^ Despite these important advances enabled by click chemistry, many challenges remain. For example, the presence of two surface-accessible cysteine residues resulted in product heterogeneity for both EDC and click chemistry. Both conjugation strategies with silk fibroin resulted in side products and β sheet formation. Therefore, more work is required to ensure the fidelity of the final silk biomolecule conjugates.

The prior state-of-the-art for polymer protein conjugates can inform conjugation strategies for silk and subsequent bench marking. However, none of the silk–asparaginase conjugate products^68,69^ were compared to the clinical benchmark PEGasparaginase (Oncaspar). The absence of *in vivo* pharmacokinetic studies further complicates the assessment of the silk–protein conjugates. Oncaspar is typically administered to patients via intramuscular injections, although intravenous infusion is also possible. Any silk conjugate would be expected to match these routes of administration. However, translating asparaginase–silk protein conjugates^68,69^ will be challenging, and similar considerations apply to the Fibroblast Growth Factor 2 conjugates.^75^ The short shelf life of liquid silk and its inherent desire to self-assemble complicate the manufacture and formulation, as well as the physical and chemical stability (ultimately impacting the robustness of the supply chain). Even overcoming these challenges still leaves asparaginase–liquid silk protein conjugate formulations posing a risk of adverse clinical reactions due to self-assembly and potential precipitation in the blood, which could ultimately trigger a thrombolytic event.

#### 4.1.2 Enzyme-Mediated Chemistries for Bioconjugation

**Figure 4:**
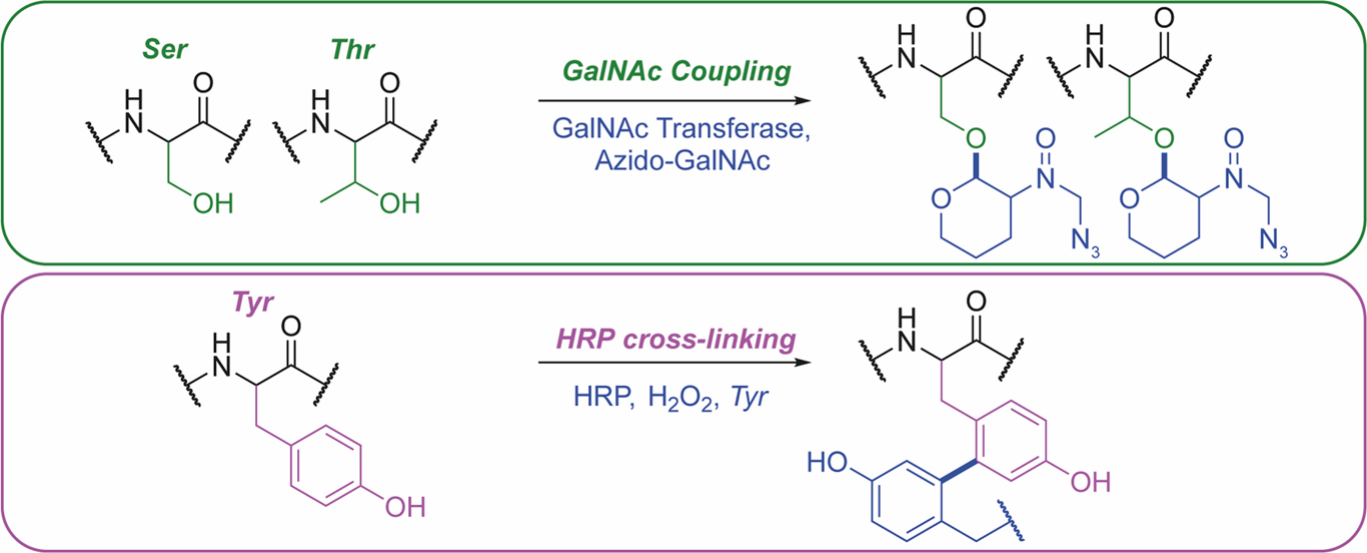
Enzyme-mediated bioconjugation techniques which display site-specificity and utilize the silk fibroin natural amino acids. The reaction schemes have been adapted from refs (41 and 78).

A spectrum of chemically modified silks has been synthesized using enzymes (e.g., tyrosinase,^58^ glutathione,^76^ laccase, genipin, horseradish peroxidase, O-GalNAc-transferases, sortase A).^5^ However, only sortase A,^77^ O-GalNAc-transferases,^78^ and horseradish peroxidase^79^ have been used to create silk bioconjugates (Figure 4). Enzymes are particularly suitable for protein modification because they are highly selective, and the catalysis is performed under physiological conditions, resulting in a high yield and a homogeneous product. Enzyme-mediated conjugation has further opened up a new chemical space, as enzymes can target specific protein sequences or glycans, which can be exploited for PEGylation and which are not readily modified by traditional chemistry approaches. For example, glycopegylation technology is used for several commercial medicines (i.e., Rebinyn, lipegfilgrastim, and Esperoct). However, silk fibroin degumming typically removes all posttranslational modifications, so glycans cannot be used as conjugation sites, although they can be reintroduced.

Enzyme-mediated conjugation of silk substrates has been used to create azide-functionalized serines.^78^ Here, liquid silk was exposed to O-GalNAc-transferases using azide-modified UDP-GalNAc sugar as the substrate. The O-GalNAc-transferases catalyze the transfer of N-acetylgalactosamine from UDP-GalNAc to the hydroxyl of serine to form O-linked glycans. Whether silk fibroin carries O-glycans naturally that could take part in the conjugation is not known; however, the degumming process is likely to remove any O-glycans should they be present. The O-GalNAc-transferase family contains 20 different enzyme types, with the classical types targeting serine residues that are located in close proximity to proline. However, of 5263 amino acids comprising the silk heavy chain, 635 are serines (12 mol %) but only 20 are prolines. Therefore, O-GalNAc-transferases with expanded specificity that tolerate proline-devoid sequences were used here to maximize the azido-GalNAc-functionalization of silk.^78^ Importantly, this study demonstrates that silk fibroin can be modified using O-GalNAc-transferases even though it is not a native target. The exact serine acceptors were not identified because the presence of too many potential acceptors in the serine-rich silk stretches prohibits meaningful mass spectroscopy analyses. Instead, click chemistry with a fluorescent reporter dye was used.

Horseradish peroxidase is used to functionalize *B. mori* silk. For example, liquid silk fibroin was covalently decorated with custom-made peptides containing either a single or triple GAGAGA or GGKGGK sequence and capped with tyrosine at either one or both ends.^79^ Horseradish peroxidase was added to the silk peptide mix to produce dityrosine cross-linking and trigger a solution–gel transition. The use of isotope-labeled peptides allowed mass spectrometry fragments to distinguish between silk backbone cross-linking, peptide cross-linking, and silk peptide functionalization. Silk backbone cross-linking dominated, followed by peptide silk functionalization. One challenge was to eliminate the unreacted peptide and cross-linker that continued to leach from the silk hydrogel.^79^

### 4.2 Heterogeneous Reactions

Silk fibroin can be processed into a broad range of material formats, including films, sponges, hydrogels, or fibers.^7^ These systems have been extensively explored for biomedical applications; late-stage functionalization of these silk formats can simplify purification, ensure modification of the reactive groups present at the material surface, improve performance, and open up new applications.^2,80^

#### 4.2.1 Silk Fiber Substrates

Both engineered and native spun fibers can be functionalized to create bioconjugate substrates. The latter approach is particularly attractive because it capitalizes on the exquisite silk fiber attributes and builds on the commercial product pipeline (e.g., DermaSilk). For example, chondroitin sulfate-decorated silk fibers were used to guide chondrocytes.^81,82^ The surface of *A. mylitta* silk was functionalized with chondroitin by first oxidizing chondroitin sulfate with sodium periodate, followed by the reaction of the oxidized chondroitin sulfate with silk to form a Schiff’s base. While experimental proof for conjugation was provided via NMR and FTIR, an assessment of the grafting density was absent. Next, silk fibers were crisscross aligned, and the hollow center was filled with a self-assembled silk hydrogel to create a three-dimensional annulus fibrosus tissue mimetic. These constructs used chondroitin sulfate-functionalized silk fibers because chondroitin sulfate is a key player in disc morphogenesis, extracellular matrix deposition, and cartilage morphogenesis and maturation. These decorated and arranged fibers modulated cell metabolism and extracellular matrix gene and protein expression while enhancing the chondrocyte differentiation potential.^81,82^ The functionalized scaffolds showed improved mechanics over time due to cell maturation and extracellular matrix deposition. *A. mylitta* silk is particularly well suited to create an annulus fibrosus tissue mimetic because this silk, unlike *B. mori* silk, contains the fibronectin-derived RGD peptide sequence that enables integrin-mediated cell–substrate attachment. Chondroitin sulfate surface decoration itself had no significant impact on the amount of matrix deposition when compared to unmodified controls.^81,82^ This indicated that this system requires further optimization to maximize its full potential. While preclinical studies using *A. mylitta* silk are promising,^36^ this silk type is currently not approved for clinical use in humans, complicating its translation to market.

#### 4.2.2 Silk Film Substrates

Chemical modifications of *B. mori* silk to modulate cell–substrate interactions are common and often use carbodiimide chemistry. However, only 3% of the total amino acid content in *B. mori* silk is aspartic and glutamic acids that carry carboxylic acid side chains available for carbodiimide chemistry, so chemical modification is limited.^41,49^ The work by Vartika Dhyani and Neetu Singh set out to increase the number of surface-accessible carboxylic acid groups.^83^ Solution-stable silk films were chemically modified by plasma etching and then surface modified using either acrylic acid or poly(2-hydroxyethyl methacrylate). The acrylic acid-modified silks were functionalized with PEG using EDC / NHS chemistry. The poly(2-hydroxyethyl methacrylate) crafting increased by 3-fold the number of reactive carboxylic acid groups, but the actual amount of surface-functionalized acrylic acid was not reported. Water contact angle measurements showed that the degree of functionalization could be tuned to impact surface hydrophobicity, with the greatest hydrophilicity for poly(2-hydroxyethyl methacrylate) grafted silk. The poly(2-hydroxyethyl methacrylate)-grafted silk fibroin surfaces showed improved cell attachment and proliferation that rivaled the performance of plasma-treated polystyrene (i.e., tissue culture plastic).^83^ This is an important observation, because tissue culture plastic has been optimized for cell culture. Cells are not able to employ integrin binding directly with either native polystyrene or *B. mori* silk surfaces. Cell–substrate engagement requires the adsorption of biopolymers from the cell culture medium (e.g., fibronectin).^84^ Protein adsorption assays showed that poly(2-hydroxyethyl methacrylate)-grafted silk increased protein adsorption to create an interface for rapid cell attachment.^83^ Overall, this work demonstrates that chemical modification of aspartic and glutamic acid groups is a viable strategy for surface modification of silks. This chemistry has also been used to surface decorate silk films with acrylic acid and phosphates, which induced mesenchymal stem cell differentiation into chondrocytes and osteoblasts, respectively.^85^

The Burke lab reports an alternative strategy to enrich hydroxyl groups on silk film surfaces by targeting tyrosine, lysine, serine, cysteine, histidine, and arginine.^54,86^ Here, silk fibroin films were decorated with hydrophilic and zwitterionic polymer brushes using atom transfer radical polymerization rather than ready-made polymers.^86^ The initiating monomers were covalently attached to the silk fibroin surface, and the polymer chains were grown from the surface up using acrylate monomers. The need arose to increase the number of surface hydroxyl groups to facilitate initiating molecule binding and subsequent polymer growth; therefore, the silk fibroin surface was enriched with hydroxyl groups by exploring two methods: ethylene oxide conjugation or a two-step oxidation reaction (Figure 3). The advantage of the ethylene oxide method is that this reaction is performed in the absence of a solvent and is thus suitable for water-soluble amorphous silk films which would otherwise dissolve in aqueous conditions (or change their secondary structure; e.g., exposure to alcohol). The two-step oxidation reaction involved exposure of the silk films to aqueous ammonium persulfate and then to ultraviolet light to promote the formation of OSO_3_^–^ groups that were hydrolyzed to carboxylic acid groups (N.B., the UV light can also increase β sheet content changing the secondary structure). This reaction can generate a large number of hydroxyl groups due to the substantial number of reactive sites, including secondary amines, along the silk protein backbone. Albumin was used as a model protein to mimic material fouling because albumin is abundant in plasma and one of the first surface-adsorbed proteins. While oxidized silk had a lower contact angle (30°) compared to unmodified silk films (contact angle 65°), no significant difference was noted in the surface adsorption of bovine serum albumin. Pegylated surfaces showed a significant reduction in albumin adsorption, whereas zwitterionic surfaces showed 2-fold less protein adsorption compared to PEGylated surfaces. Cell attachment and spreading were also impacted and followed the same pattern seen for albumin adsorption.^86^ This is not surprising because protein surface adsorption is important for providing a cell–material interface for cell attachment via integrins.

#### 4.2.3 Silk Scaffold Substrates

Bioconjugated silk substrates are also capable of tuning biology. For example, chemically modified *B. mori* silk with surface-conjugated lactose was synthesized to modulate fibroblast biology.^87^ Silk fibroin films and three-dimensional scaffolds were surface decorated using cyanuric chloride-activated lactose to predominately target tyrosines and lysines (Figure 3). These substrates were then used for two- and three-dimensional culture of primary subcutaneous fibroblasts. The modified substrates improved cell attachment over the untreated controls. The lactose substrates were able to modulate fibroblast biology by minimizing the de novo development of a myofibroblast phenotype and were even able to trigger fibrogenic myofibroblasts to dedifferentiate back into resting fibroblasts. This effect was attributed to the presence of available galactose residues that enabled lectin-mediated cell binding and signaling.^87^ These observations open up the possibility of using these materials to steer other mesenchymal cell lineages away from a pathological phenotype.

An interesting concept is the scaffold-based traps for *in vivo* use^88^ or sample cleanup.^89^ In the latter case, regenerated *B. mori* liquid silk was lyophilized and scaffolds were functionalized with covalently binding capture tags.^89^ The resulting sponge was treated with ethanol to induce beta sheets and a surface decorated with the poly-specific microbial targeting molecule apolipoprotein H. Apolipoprotein H is a glycoprotein that circulates in the blood and displays the characteristic cationic amino acid sequence KNKEKK toward the C-terminus. Apolipoprotein H is an ideal capture protein because it strongly interacts with negatively charged phospholipids (e.g., lipopolysaccharides, endotoxins, and membrane proteins of microorganisms). The recombinant apolipoprotein H was engineered with an additional pentatyrosine at the N-terminus, which was used for covalent silk coupling via horseradish peroxidase-mediated dityrosine cross-linking. Conjugated apolipoprotein H showed improved activity, probably due to the optimized spatial arrangement via N-terminal immobilization and the microbial capture motif at the C-terminal domain. The resulting apolipoprotein silk scaffold was able to trap microbial model compounds, including bacteria and viruses, from laboratory suspension samples.^89^ Pushing these systems up to the Technology Readiness Level will be an important task for laying the groundwork for similar platforms.

#### 4.2.4 Silk Nanoparticles

Silk particles are now often proposed for use in drug delivery applications (reviewed in ref (90)). One motivation is to improve drug targeting. The physicochemical properties of the free drug determine its pharmacokinetic characteristics (e.g., plasma protein binding, tissue distribution, subcellular trafficking, metabolism, elimination, etc.). However, a well-designed particle formulation will override these physicochemical hallmarks, ultimately dictating pharmacokinetic characteristics that are engineered by design.^91^ For example, drug-loaded nanoparticles are often proposed for solid tumor targeting, with a number of these systems routinely used in the clinic.^92^ These formulations eliminate the need for toxic solvents to solubilize the drug, reduce drug exposure to healthy tissues, and improve solid tumor targeting. Leaky blood vessels of solid tumors, coupled with reduced lymphatic drainage, enhances tumor tissue permeation and retention (known as the EPR effect, first proposed by Yasuhiro Matsumura and Hiroshi Maeda in 1986^93^). This concept has sparked renewed interest due to tumor heterogeneity and variable patient responses.^94^ We^91^ and others^90,95,96^ have previously reviewed emerging silk nanomedicines. Improved manufacturing tools^43,97−99^ and model systems^100^ help us to better understand critical quality attributes of silk nanoparticles. One key goal when designing silk nanoparticles for drug delivery is to improve cellular drug uptake and intracellular drug delivery. Receptor-mediated endocytosis is one strategy that can be used to achieve this.

The first chemically modified nanoparticle designed for receptor-mediated uptake was reported by Subhas Kundu and colleagues.^101^*A. mylitta* silk was nanoprecipitated in acetone into uniform 200 nm nanoparticles that were stable and spherical. These particles were then suspended and functionalized with folate using EDC NHS coupling chemistry. While the surface density of conjugated folate is unknown, these functionalized particles showed improved receptor-mediated uptake into human MDA-MB-231 triple-negative breast cancer cells. Loading of these functionalized silk particles with the anticancer drug doxorubicin using solution-based adsorption was most effective with the folate targeted nanoparticles at equivalent drug doses. *A. mylitta* silk nanoparticles showed pH-dependent doxorubicin release that was fastest at acidic pH,^101^ with similar trends observed with *B. mori* silk.^102^

Responsiveness to pH is a useful trigger for drug release, both in the acidic tumor microenvironment (approximately pH 6.5 to 6.9) and for intracellular lysosomotropic drug release (approximately pH 4.5). Dedicated wet-lab experiments with simulated organelle pH values showed pH responsiveness^102,103^ but also degradation-dependent release,^18^ while single-cell microscopy of live cells confirmed lysosomotropic drug delivery.^104^ Molecular modeling showed that ionizable amino acid residues, especially glutamic and aspartic acids, are responsible for the pH-dependent doxorubicin–silk interaction in *B. mori*.^100^ These principles are likely to be important for *A. mylitta* silk as well, based on sequence homology and particle attributes.

Silk-based nanomedicines proposed for systemic administration and solid tumor targeting require “stealth” design principles to maximize their pharmacokinetic parameters and improve tumor targeting.^103^ PEGylation influences the pharmacodynamics of drugs and drug delivery systems. For example, PEGylation stabilizes colloidal drug carriers: recent examples are PEGylated lipid nanoparticles carrying mRNA payloads as coronavirus vaccines (e.g., Spikevax and Comirnaty by Moderna and BioNTech & Pfizer, respectively).^105^ Established cyanuric chloride conjugation chemistry was used for silk, resulting in a 20% grafting efficiency. PEG was surface grafted to preformed silk nanoparticles using cyanuric chloride-activated methoxypoly(ethylene glycol) (5000 g/mol). First-generation PEGylated silk nanoparticles showed improved colloidal stability, with an increase in their hydrodynamic radius and a more neutral zeta potential. These PEGylated silk nanoparticles could be readily loaded by drug adsorption from solution, and doxorubicin- and propranolol-loaded nanoparticles were used in combination and showed a synergistic therapeutic anticancer response *in vitro*. PEGylated silk nanoparticles showed improved colloidal stability,^103^ while macrophages exposed to unmodified and PEGylated silks showed different pro-inflammatory and metabolic responses.^29^ Dedicated solution-based silk studies showed that cyanuric chloride-activated methoxy-PEG reacted with the phenolic hydroxyl group of the tyrosine residue, in addition to the ε-amino group of lysine and the imidazole group of histidine residues present in silk fibroin.^106^ The degree of modification was greater than 3.3 mol %, which is good based on the lysine (0.32 mol %), histidine (0.18 mol %), and tyrosine (5 mol %) contents.^106^ A limitation is that this insight relates to solution-based silks, and more work is required to confirm this in solid-state silk conjugates.

Silk nanoparticles decorated with targeting ligands are typically loaded either postsynthesis (e.g., refs (101 and 107)) or in situ^108^ to improve drug delivery. For example, in situ drug loading was performed by adding paclitaxel in ethanol to aqueous 0.5% (w/v) silk fibroin under stirring, resulting in 10% weight/weight loading.^108^ After stirring for 5 min, the resulting suspension was centrifuged and the drug-loaded silk particles were collected. The particles were then surface decorated using EDC-NHS conjugation to form a dual functional peptide, anti-EGFR–iRGD, with a reported conjugation efficiency in excess of 75%. Anti-EGFR–iRGD consists of an anti-epidermal growth factor receptor variable domain derived from a heavy chain of the antibody fused to a cyclic nona-RGD peptide. This recombinant protein dually targeted EGFR and α_v_β_3_/α_v_β_5_ integrins, resulting in overall improved anticancer efficacy both *in vitro* and *in vivo.* At 12 to 72 h after injection into tumor-bearing mice, the targeted particles showed substantially higher tumor targeting compared to controls, while the overall tumor growth was reduced 2-fold with targeted particles compared to paclitaxel control particles.^108^ Nonetheless, the absence of a freely diffusible paclitaxel control group or soluble targeting residue decoys to prove receptor engagement complicates the interpretation of the *in vivo* data.

Improving insulin delivery has been widely explored, including studies using silk as a carrier material to achieve long-acting insulin formulations that reduce dose frequency. One strategy involves particle-mediated delivery by silk nanoparticles assembled into high-crystallinity, 40–120 nm sized particles by acetone nanoprecipitation.^109^ These particles were surface decorated with insulin using 0.7% glutaraldehyde cross-linking for 8 h and an insulin-to-silk fibroin ratio of 30 IU and 15 mg, respectively. The use of particles, rather than solution-based systems, simplified product cleanup. Exposure of the conjugate to isolated human serum samples improved insulin stability by increasing the “plasmatic” half-life from 17 to 42 h. Exposure to trypsin also confirmed an increased proteolytic stability of up to 40%.^109^ However, all the observations were based on ELISA measurements rather than functional assays. No *in vitro* or *in vivo* pharmacological assays were performed, so the actual effectiveness of this formulation for blood glucose control is unknown. One potential caveat is the need for breakage of stable covalent bonds for the release of the immobilized insulin without compromising insulin activity. The design of a zero-length biologically responsive linker could navigate this challenge while potentially simplifying product characterization and critical quality attributes.

## 5 Emerging and Site-Specific Bioconjugates

### 5.1 Recombinant Spidroins

Recombinant protein expression provides a near endless array of designer proteins, while advances in expression technologies now also provide the toolbox required to create proteins with noncanonical functionality. Comprehensive reviews of spider silk-inspired proteins and their design and applications have been detailed previously.^3,4,110^

Recombinant silk-inspired silks are particularly well suited for tailored applications, including coatings for medical use. Seminal work by the Hedhammar lab designed biologically active silk coatings to improve orthopedic and dental implant performance by reducing bacterial infection and encouraging bone ingrowth via fibronectin-mediated integrin engagement.^77^ A recombinant partial spider silk-like protein was fused with a fibronectin cell binding domain, applied to test surfaces, and functionalized with one of the following biologically active enzymes: dispersin B (DspB, a biofilm disrupter), PlySs2, or SAL-1 (endolysins). These payloads were expressed recombinantly and had a sortase recognition tag to permit site-specific conjugation to silk using transpeptidase sortase A. Silk coatings functionalized with DspB or the endolysins showed reduced bacterial adhesion compared to silk controls. These enzymes did not reduce bacterial viability but interfered with bacterial substrate adhesion, resulting in “mobile” bacteria.^77^

First-generation spider silk-inspired proteins, including major ampullate spidroin from the European garden spider *Araneus diadematus*, have been used for click chemistry. Thomas Scheibel and colleagues pioneered this technology and surface modified silk films by first using EDC/NHS conjugation of alkyne-terminated azidopropylamine.^111^ Azide–alkyne cycloaddition was then performed to surface conjugate glycopolymers (molecular weight range from 10 to 30 kg/mol). These glycopolymer-decorated films showed decreased water contact angles, increased extracellular matrix adsorption from solution, and improved cell adhesion.^111^ In a separate study, these silk-inspired proteins were chemically modified at the N-terminus to yield azide functionality.^112^ These studies required chemical modifications conducted after protein expression.

**Figure 5:**
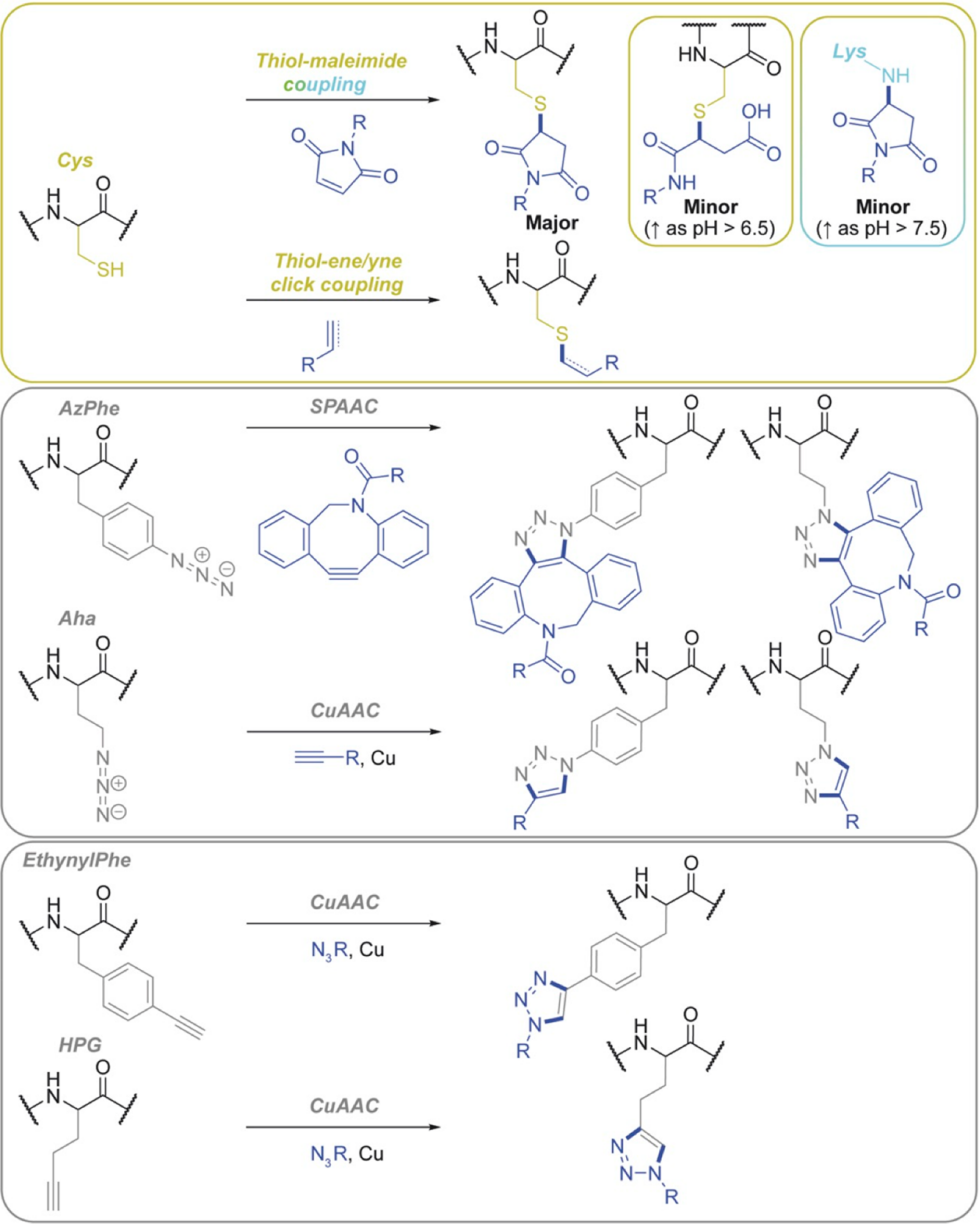
Bioconjugation of recombinant silk fibroin using bioorthogonal thiol–maleimide, thiol–ene, and azide–alkyne click chemistry. Reaction schemes have been adapted from refs (40 and 117−120).

For second-generation silks, Thomas Scheibel, and colleagues extended the coding sequence of the N-terminal end with a short amino acid tag consisting of 14 residues containing a single cysteine (GCGGSGGGGSGGGG), thereby introducing the only cysteine into the recombinant silk^113^ (reviewed in ref (110)). This cysteine was targeted for thiol–ene click chemistry (Figure 5), which achieved a coupling efficiency of 70–90%, as assessed with a fluorescent tracer. This engineered silk was then explored for potential applications. For example, silk was templated with titanium dioxide and gold to create a photocatalyst to generate hydrogen (for silk power generators^114^). The gold patterning exploited thiol–ene chemistry using maleimide-functionalized gold nanoparticles.^115^ Gold functionalization also enables the use of these system for biomedical applications (e.g., sensors, thermal therapy, etc.). In a different study, electrically conducting Janus fibers were produced.^116^ The modified silk was cospun with unmodified silks to create Janus-like silk fibers with click functionality for maleimide-functionalized gold thiol–ene chemistry via a Michael addition reaction.^116^ However, cysteine maleimide conjugates can undergo exchange reactions, resulting in the deconjugation of the functional molecule. This raises concerns regarding the long-term stability of these conjugates, especially *in vivo*. None of these strategies used noncanonical amino acids, and the use of a single cysteine per silk molecule reduces the possible degree of functionalization.

**Figure 6:**
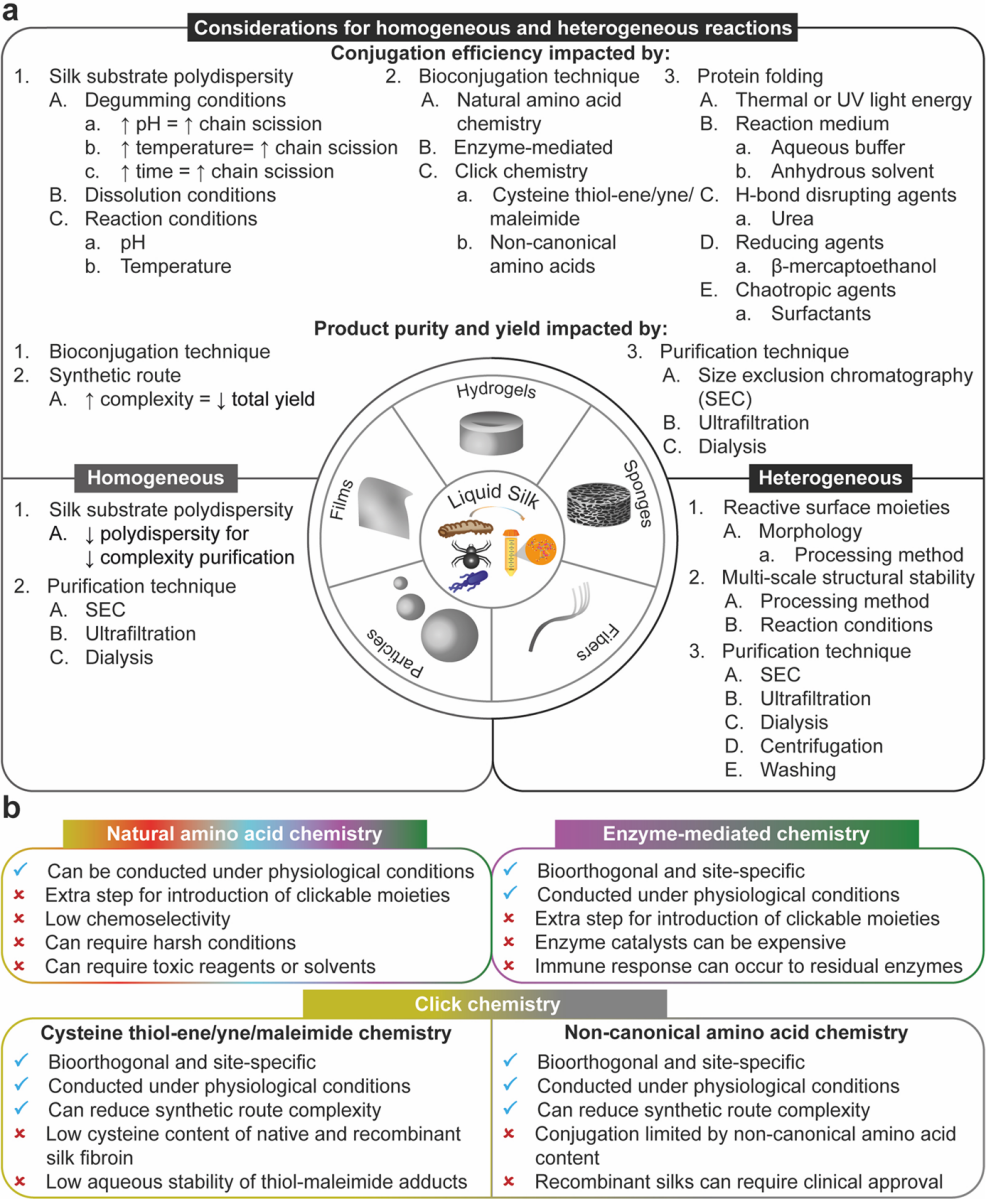
(a) Selected considerations for the bioconjugation of silk fibroin using homogeneous and heterogeneous reactions and (b) selected advantages and disadvantages of the bioconjugation techniques discussed herein.

Third-generation silks contain a bioorthogonal noncanonical amino acid tag which provides an advantage over native silks by enabling site-specific chemical modification in one step and without the addition of enzymatic catalysts (Figures 5–6). Seminal work by Sara Goodacre, Neil Thomas, and colleagues used synthetic biology to express spider silk-inspired proteins (i.e., a 37 kDa “mini-spidroin”) with click chemistry functionality already encoded within the protein.^40^ This mini-spidroin comprised four repetitive polyalanine blocks flanked by five glycine-rich amorphous linkers and a serine–alanine domain adjacent to the nonrepetitive C-terminal domain. The C-domain contained two methionines. This sequence was expressed in a methionine auxotrophic DL41 *Escherichia coli* able to handle noncanonical methionine amino acids. Methionine was substituted for l-azidohomoalanine, which was subsequently incorporated into the C-terminal domain of the mini-spidroin. Successful click chemistry was demonstrated using fluorescent markers, while biological functionality was provided with the antibiotic levofloxacin. However, the degree of methionine substitution with l-azidohomoalanine was not quantified. Unlike previous studies that incorporated functionality into mini-spidroins via the expression of specific amino acid sequences or chemical modification of the protein postexpression, this work introduced l-azidohomoalanine via bioorthogonal noncanonical amino acid tagging.^40^ This tagging approach enables bespoke click chemistry that is bioorthogonal because the reactive groups are not found in nature. Importantly, the simplified synthetic routes imparted by using third-generation silk compared to natural silk substrates can potentially reduce the cost of purification and increase product yields (Figure 6).

Finally, advances in protein expression have also enabled the creation of native-sized spider-like proteins (250–320 kDa) through the use of metabolically engineered *Escherichia coli* to elevate the glycyl-tRNA to process these glycine-rich sequences.^121^

### 5.2 Recombinant *Bombyx mori* Silk Fibroin

The use of *in vitro* expression hosts with mutant tRNA synthetases that have expanded substrate specificity now also enables the creation of recombinant silks with noncanonical amino acids. These systems were first pioneered by Hidetoshi Teramoto and colleagues *in vitro* in genetically modified *B. mori* cell cultures,^122^ and they subsequently translated this work to sericulture to produce silks with bioorthogonal noncanonical amino acid tags (detailed below). Nonetheless, all these novel silks will require rigorous safety assessment, while lessons learned with other bioconjugates must inform our silk research. For example, a recombinant interferon beta with a bioorthogonal noncanonical amino acid tag used for PEGylation was subjected to a human Phase I clinical trial but showed higher anti-PEG immunogenicity than Plegridy (PEG-IFN beta), so the trial was stopped.^123^ However, PEGylated bovine granulocyte colony-stimulating factor is clinically approved for veterinary use (PEGbovigrastim; Imrestor). Here, a bioorthogonal noncanonical amino acid tag is used for PEGylation.^124^

The domesticated *B. mori* silkworm is an exquisite protein expression host with the capacity to produce large amounts of silk fibroin (approximately 400 mg). In the last and final fifth instar, the silkworm synthesizes the majority of the silk over only a few days and stores this silk I in the silk gland until it starts to continuously spin its cocoon over approximately 24 h. Silkworms have been repurposed for the expression of experimental and therapeutic proteins (reviewed in ref (125)). Interferon produced by *B. mori* silkworms was the first product marketed in Japan in 1993 by Toray Industries Inc. for the treatment of feline calicivirus infection and subsequently approved in the EU in 2001; this product is now also approved for canine parvovirus infection. This clinical interferon product is not a bioconjugate but is expressed in the middle silk gland under a sericin promotor and incorporated into the sericin coating encasing the silk filaments. However, this clinical translation demonstrates that *B. mori* can be used as an expression host that can navigate the regulatory pathway for clinical approval.

Preclinical studies have engineered the silkworm genome to express a protein of interest within the silk fibroin sequence. For example, Mitsuru Sato and colleagues developed a silkworm expression system for a single-chain variable fragment against Wiskott–Aldrich syndrome protein.^126^ This protein is associated with an X-linked recessive disease characterized by immune dysregulation and microthrombocytopenia. Germline modification of *B. mori* was performed to introduce the single-chain variable fragment into the genome using piggyBac transposase and a fluorescence DsRed2 selection marker. Specifically, the therapeutic protein was inserted downstream of the silk light chain-coding cDNA under the control of the fibroin L-chain promoter to minimize potential negative consequences on overall silk production. Fertilized silk eggs were microinjected with the plasmid, and the eggs were allowed to develop into moths that were then crossed. The G1 broods were screened for transgenic individuals (4% success rate), and the selected individuals, identified using the DsRed2 selection marker, were reared and allowed to spin their cocoons. Three grams of cocoons were chopped and dissolved in 9 M LiBr at 37 °C, dialyzed against water, and lyophilized. SDS-PAGE analysis of the dried powder showed the expression of the therapeutic protein as a silk fibroin light-chain conjugate in the absence of protein degradation. The single-chain variable fragment approach is better than expressing the full-length antibody because the fragments often retain their biological activity even under strong reducing conditions, have a short amino acid sequence, and show resilience when incorporated into a protein scaffold. Using *B. mori* as an expression host has several advantages over the traditional antibody production process, because it reduces the number of processing steps and has the potential to produce larger amounts of protein. Here, sample analysis showed that approximately 25% of the silk fibroin light chain was expressed in the therapeutic target.^126^ The *B. mori* gland has evolved as a highly efficient “protein factory” and is therefore poised for recombinant protein production.

Hidetoshi Teramoto and colleagues genetically engineered *B. mori* to carry a mutant phenylalanyl-tRNA synthetase with expanded substrate recognition capabilities. *In vivo* feeding of these silkworms with *p*-chloro-, *p*-bromo-, and *p*-azido-substituted analogues of l-phenylalanine resulted in their incorporation into spun silk fibers.^118^ This innovative system has since been optimized to increase the substitution of *p*-azido-substituted phenylalanine,^127^ which enabled silk scale-up.^128^ A photostable ethynylphenylalanine-modified silk was also developed using phenylalanyl-tRNA synthetase modified silkworms to simplify silk processing.^119^ Silkworms with canonical methionyl-tRNA synthetase successfully incorporated homopropargyl-glycine and azidohomoalanine that targeted the single methionine of the silk light chain and the three methionines at the N-terminus of the silk heavy chain.^117^ Although the azido and alkyne content of these silks is low, these azido and alkyne groups have subsequently been used to functionalize silk fibers, films, and scaffolds with fluorescent tracers, patterns, polymers, or biotin ligands for downstream applications.^118,128−130^ Overall, these seminal studies demonstrate the utility of genetic code expansion to create novel silks suitable for bioconjugation.

## 6 Conclusions and Outlook

This perspective highlights silk bioconjugate developments, including bioconjugated substrates, to enhance cell–material interactions, bioconjugated silk particles, and silk–protein conjugates. To date, none of these products have entered routine clinical use. Silk standardization and the definition of critical quality attributes are key factors for enabling more products to complete their journeys from bench to bedside;^5^ therefore, a consensus silk framework would be beneficial that will also impact silk bioconjugates. Ultimately, this effort will expand the numbers of clinical trials (currently <40) and clinically approved *B. mori* products (currently approximately 4).^2,5^ The silk field is now at a critical juncture and bears some similarity to the nanomedicine field of the early 2000s^131,132^ that ultimately delivered mRNA vaccines during the SARS-CoV-2 pandemic. Innovative ideas and tools, as well as clever marketing, have propelled silk to “stardom” and raise hope. Now is also the time to deliver on these promises and moderate what can be achieved to safeguard the field and, most importantly, patients. Throughout this perspective, open questions and areas that require more work are highlighted. However, a state-of-the-art molecular toolbox enabling site-specific conjugation, recombinant expression, bioorthogonal noncanonical amino acid tagging, and genetic engineering *in vivo* is critical for translating next-generation research into products. The production of interferon using genetically engineered *B. mori* silkworms (Toray Industries, Inc.) serves as an excellent example of how innovative products can progress to the market, albeit deviating from the silk bioconjugate paradigm explored here. Overall, this perspective highlights a domain of new “sunrise” capabilities and development opportunities for silk bioconjugates that may offer new ways of delivering improved healthcare.
